# Low Protein Intake Is Associated with the Risk of Functional Impairment in Older Adults in an Age- and Gender-Specific Manner: A SHARE-Based Study

**DOI:** 10.3390/nu18071058

**Published:** 2026-03-26

**Authors:** Rizwan Qaisar, M. Azhar Hussain, Salma Naheed, Khalid Saeed, Asima Karim, Firdos Ahmad, Sandra Haider, Maha H. Alhussain, Shaea A. Alkahtani

**Affiliations:** 1 Department of Basic Medical Sciences, College of Medicine, University of Sharjah, Sharjah 27272, United Arab Emirates; rqaisar@sharjah.ac.ae (R.Q.); akarim@sharjah.ac.ae (A.K.); fahmad@sharjah.ac.ae (F.A.); 2 Space Medicine Research Group, Research Institute for Medical and Health Sciences, University of Sharjah, Sharjah 27272, United Arab Emirates; 3 Cardiovascular Research Group, Research Institute for Medical and Health Sciences, University of Sharjah, Sharjah 27272, United Arab Emirates; 4 Department of Finance and Economics, College of Business Administration, University of Sharjah, Sharjah 27272, United Arab Emirates; mazhar@sharjah.ac.ae; 5 Department of Social Sciences and Business, Roskilde University, DK-4000 Roskilde, Denmark; 6 Shifa College of Medicine, Shifa Tameer-e-Millat University, Islamabad 44000, Pakistan; 7 The In Silico Drug Discovery Group, University of Helsinki, 00170 Helsinki, Finland; 8 King Abdullah International Medical Research Center, King Saud bin Abdulaziz University for Health Sciences, Riyadh 11451, Saudi Arabia; 9 Iron Biology Research Group, Research Institute for Medical and Health Sciences, University of Sharjah, Sharjah 27272, United Arab Emirates; 10 Department of Social and Preventive Medicine, Center for Public Health, Medical University of Vienna, 1090 Vienna, Austria; 11 Department of Food Sciences and Nutrition, College of Food and Agricultural Sciences, King Saud University, Riyadh 11451, Saudi Arabia; mhussien@ksu.edu.sa; 12 Exercise Physiology Department, College of Sport Sciences and Physical Activity, King Saud University, P.O. Box 1949, Riyadh 11451, Saudi Arabia

**Keywords:** protein intake, handgrip strength, physical function, aging, SHARE dataset

## Abstract

**Background and Objectives:** Functional decline and sarcopenia are major aging-related concerns. While protein intake is known to influence muscle health, its longitudinal impact on strength and physical function across age and gender remains underexplored. We assessed whether low protein intake correlate with future onset of low handgrip strength (HGS) and physical impairments in older adults using SHARE data. **Methods:** We analyzed 38,073 adults aged ≥50 years from 27 European countries using SHARE Waves 8 (2019/20) and 9 (2021/22). A protein intake index was derived from the frequency of consuming dairy, legumes/eggs, and meat/fish/poultry. Low intake was defined as the lowest decile. Logistic regression models, adjusted for age, gender, country, and baseline health, examined associations with low HGS and ten physical difficulties, stratified by age (50–65 vs. ≥66 years) and gender. **Results:** Low protein intake is associated with higher odds of low HGS in men (OR = 1.39 for 50–65; OR = 1.35 for ≥66) and older women (OR = 1.21). It was also associated with higher odds of mobility-related limitations, including walking 100 m (ORs = 1.25–1.53), stooping/kneeling (ORs = 1.20–1.19 in women), and reaching overhead (ORs = 1.19–1.33). Strength-related tasks, such as pushing/pulling large objects were more affected in men (ORs = 1.44 and 1.21). Notably, women aged 50–65 had over twice the odds of toileting difficulty (OR = 2.27) and significantly higher odds of difficulty shopping (OR = 1.65). These patterns highlight gender- and age-specific vulnerabilities. **Conclusions:** Low protein intake is associated with modest but consistent increases in the risk of reduced muscle strength and functional difficulties in older adults. Tailored nutritional strategies may mitigate age- and gender-specific risks to physical independence.

## 1. Introduction

Functional impairments are a common consequence of aging, characterized by a progressive decline in the ability to perform everyday tasks, such as walking, climbing stairs, rising from a chair, and maintaining balance [1]. These limitations not only reduce independence and quality of life but also increase the risk of falls, hospitalization, and long-term care placement [1]. Functional decline is multifactorial, involving musculoskeletal deterioration, neurological changes, and nutritional deficiencies [2]. Among the various indicators of physical function, handgrip strength (HGS) has emerged as a robust and easily measurable biomarker, as a low HGS is associated with poor mobility and higher mortality risk [3].

In addition to HGS, difficulties in performing basic activities of daily living (ADLs) such as bathing, dressing, and toileting are early signs of physical decline. These impairments often precede more severe disability and are predictive of adverse health outcomes [4]. The ability to rise from a chair, walk short distances, and climb stairs is particularly relevant in assessing functional status in older adults. These tasks require coordinated neuromuscular function and are sensitive to changes in muscle strength and endurance [1,4].

Sarcopenia, defined as the age-related loss of skeletal muscle mass and strength, is a major contributor to functional impairment in older adults. It is associated with increased risks of frailty, falls, and mortality [5]. The European Working Group on Sarcopenia in Older People (EWGSOP2) recommends using HGS as a primary diagnostic criterion for sarcopenia due to its substantial predictive value for adverse outcomes [3,5]. Sarcopenia is now recognized as a geriatric syndrome with significant public health implications, especially in aging societies.

Dietary protein intake is a key modifiable factor in the prevention and management of sarcopenia and functional decline [6]. Protein is essential for muscle protein synthesis, tissue repair, and immune function. In older adults, anabolic resistance reduces the efficiency of muscle protein synthesis in response to dietary protein, necessitating higher intake levels to maintain muscle mass and function [6]. The effects of dietary protein on muscle health are strongly influenced by both protein quality and physical activity. High-quality proteins rich in essential amino acids, particularly leucine, more effectively stimulate muscle protein synthesis, while regular physical activity, especially resistance exercise, acts synergistically with protein intake to preserve muscle strength and physical function in older adults [7,8,9]. Despite this, many older individuals consume less protein than recommended due to reduced appetite, dental problems, socioeconomic limitations, and comorbidities [10].

Current dietary recommendations indicate that adults under the age of 65 should consume a minimum of 0.8 g of protein per kilogram of body weight per day, whereas individuals aged 65 years and older are advised to consume at least one gram of protein per kilogram of body weight per day [11]. However, the recommended values may vary depending on the presence of underlying diseases.

A growing body of evidence from both observational and interventional research supports the role of adequate dietary protein intake in maintaining muscle mass, strength, and physical function in older adults [6]. Insufficient protein consumption has been consistently linked to accelerated muscle loss, reduced mobility, and increased vulnerability to functional impairments [12]. Conversely, higher protein intake is associated with improved physical performance, better preservation of lean body mass, and reduced risk of disability [13]. These findings underscore the importance of protein as a vital nutritional component in promoting musculoskeletal health and delaying the onset of age-related physical decline.

Despite growing interest in nutritional interventions, several gaps remain in the literature. Most studies are cross-sectional, limiting causal inference. Few studies have examined the impact of protein intake on HGS and a broad range of physical difficulties. There is limited data on how protein intake affects older men and women differently across age groups. Existing studies often rely on nutrient tracking rather than food frequency-based indices, which may be more practical and scalable in extensive population studies. Moreover, the interaction between protein intake and other lifestyle factors such as physical activity, comorbidities, and socioeconomic status is not well understood.

The Survey of Health, Ageing and Retirement in Europe (SHARE) offers a unique opportunity to address these gaps. SHARE is a longitudinal, multidisciplinary panel study that collects harmonized data on health, socioeconomic status, and social networks among individuals aged 50 and older across European countries [14]. It includes detailed information on dietary habits, physical function, HGS, and chronic conditions, making it an ideal resource for studying aging-related outcomes. SHARE data are collected through computer-assisted personal interviews and cover a wide range of domains, including demographics, living conditions, and both physical and mental health [14].

We derived a protein intake score based on the frequency of consumption of key protein-rich food groups, including dairy, legumes, eggs, and meat, fish, or chicken. We then examined whether low protein intake at baseline is associated with the future onset of low HGS and various physical difficulties, including walking, climbing stairs, rising from a chair, and performing daily tasks. Stratified analyses were conducted by age group (50–65 years versus 66 years and older) and gender to capture nuanced patterns.

We hypothesized that older adults with low protein intake are at significantly higher risk of developing low HGS and physical difficulties over time. We tested this hypothesis using longitudinal regression models adjusted for age, gender, country, and baseline health status.

## 2. Materials and Methods

All the individual level applied data is from the SHARE database that is a harmonized longitudinal panel survey conducted on respondents aged 50+ years almost entirely from European countries [14]. Interviews were conducted through computer-assisted personal interviews (CAPI) that covered various topics, including demographics, socioeconomics, living conditions, as well as physical and mental health states. The initial information came from the eighth wave of SHARE, which was fielded in 2019/2020 while the follow up information came from the nineth and thus latest available wave, which was fielded in 2021/2022. Further and many more details are available on the SHARE website [15]. The sample encompasses 27 European countries: Austria, Belgium, Bulgaria, Croatia, Cyprus, Czech Republic, Denmark, Estonia, Finland, France, Germany, Greece, Hungary, Israel, Italy, Latvia, Lithuania, Luxembourg, Malta, Netherlands, Poland, Romania, Slovakia, Slovenia, Spain, Sweden, and Switzerland. Individuals were excluded if they were incarcerated, hospitalized, or out of the country for the entire survey period, unable to speak the country’s language(s), or had moved to an unknown address, elaboration is available in [14]. SHARE respondents that were previously interviewed in the longitudinal study were later traced and re-interviewed if they relocated within the country [14].

Information on the main variable related to protein intake was obtained by the following three questions (numbered as 26, 27, and 28) on three food groups: “(Please look at card 16.) In a regular week, how often do you have a serving of dairy products such as a glass of milk, cheese in a sandwich, a cup of yogurt or a can of high protein supplement?”; “(Please look at card 16.) In a regular week, how often do you have a serving of legumes, beans or eggs?”; “(Please look at card 16.) In a regular week, how often do you eat meat, fish or poultry?”. The possible answers to each of the three questions were: “1. Every day”; “2. 3–6 times a week”; “3. Twice a week”; “4. Once a week”; “5. Less than once a week”. We transformed these frequencies into a fraction of daily intake with the values (*X_F_*): 1 (daily intake, weight is 100%), 0.6429, 0.2857, 0.1429, 0.0714 (less than once a week, weight is 7.14% = 2 × 1/7). Based on these three frequencies for the individual, a protein index was calculated with increasing weight reflecting higher protein contents: 8 g protein per 100 g food for dairy products; 12 g protein per 100 g legumes/eggs; 20 g per 100 g meat foods. Thus, the protein intake index becomes:

Protein Intake Index = 8 × *Dairy_F_* + 12 × *Legumes_F_* + 20 × *MeatsF*,

which means the minimum of the index is 2.8571 (consumes any of the three food product groups less than once weekly) and the maximum is 40 (consumes all three food product groups daily). The index was linearly transformed to the 0–100 range. Based on this protein index, low protein intake is defined as belonging to the lowest protein intake decile, i.e., the ten percent of the sampled individuals with the lowest protein intake. For interpretation, the index reflects both frequency and diversity of protein-rich foods. For example, a person consuming dairy products daily but eating legumes or eggs and meat or fish only once per week would obtain an intermediate index value, driven mainly by frequent intake of a lower protein density source. In contrast, individuals consuming all three food groups several times per week achieve higher index values due to combined intake of multiple protein dense foods. Those reporting very infrequent consumption across all groups cluster at the lower end of the index and are therefore more likely to fall within the lowest decile. This frequency-based protein intake index has not been formally validated against quantitative dietary records, which are not available in SHARE. However, similar food frequency-based indices have been widely used in large aging cohorts to rank individuals according to habitual dietary exposure and to study diet health relationships [12,16]. Lastly, the protein intake index is intended to capture relative habitual exposure rather than absolute protein intake in grams per day, as SHARE does not collect portion size or comprehensive dietary data. The weighting scheme reflects approximate differences in protein density across major food groups and was applied to rank individuals consistently within the dataset rather than to estimate exact intake. Although not all dietary protein sources are included, the selected food groups represent the primary contributors to protein intake in European older adults and are harmonized across participating countries, supporting comparability in large scale analyses.

A number of different difficulties or medical conditions were investigated. All of these are outcome variables potentially affected by protein intake as defined above.

The outcome HGS was assessed with a hand-held dynamometer. Each individual completed two trials each with their left and right hands. The maximum value recorded from these four measurements was selected for the analysis. A low HGS was determined using gender-specific thresholds from the EWGSOP2 guidelines: HGS ≤ 27 kg for men and HGS ≤ 16 kg for women [5].

The interviewed persons were asked to report if they had any difficulty doing various everyday activities. Difficulties that were expected to last less than three months at the time of responses were excluded. Among the listed activities were: “Getting up from a chair after sitting for long periods”; “Walking 100 m”; “Climbing several flights of stairs without resting”; “Stooping, kneeling, or crouching”; “Reaching or extending your arms above shoulder level”; and “Pulling or pushing large objects like a living room chair”.

The remaining outcomes are other activities were based on the question “Please tell me if you have any difficulty with these activities because of a physical, mental, emotional or memory problem. Again, exclude any difficulties you expect to last less than three months.”. Among the difficulties were: “Bathing or showering”; “Using the toilet, including getting up or down”; and “Shopping for groceries”.

All outcomes are from the follow up in wave 9, while gender, age, and protein intake are from baseline wave 8. Accordingly, all outcomes represent incident functional impairment reported at follow up, conditional on baseline exposure status. After excluding some observations due to age being less than 50 years (972 participants), missing food group intake frequency (408 participants excluded due to missing dairy information, 89 excluded due to missing legumes information, and 27 excluded due missing meat information), or body weight being less than 40 kg (2785 participants excluded), a large sample of 38,073 respondents observed in both wave 8 and wave 9 remained and was used for all analyses in the following.

### Statistical Analysis

We used multiple logit regression analysis to determine how protein intake affects outcomes related to difficulties and medical conditions. The following regression equation was applied gender-age combinations:

$$\ln\frac{\pi_{j}}{1-\pi_{j}}=\beta_{0}+\beta_{1}\cdot Protein1Decile+Country\cdot \beta_{2}+\epsilon$$

where $\pi_{j}$ represents the probability of outcome j, j = {1, …, 10} = {Low HGS, …, Difficulty shopping for groceries}. The model included a binary indicator for belonging to the lowest decile of protein intake and was adjusted for age group, gender, and country of residence using country specific dummy variables to account for cross country heterogeneity. Four set of regressions were carried out with combinations of the two genders and the two age groups below/above 66 years. The regression parameters are presented as odds $\frac{\pi_{j}}{1-\pi_{j}}$ ratios. The statistical data analyses were performed applying the STATA software package, version 19 (StataCorp LLC, College Station, TX, USA).

## 3. Results

Table 1 presents the characteristics of the sample population categorized by protein intake, distinguishing between individuals in the lowest decile of protein consumption (low intake) and those with normal intake. Among the 38,073 participants, 9.5% fell into the low protein intake group. Women comprised a larger share of the sample (57.5%) compared to men (42.5%), with slightly lower prevalence of low protein intake (9.2% vs. 9.8%, *p* = 0.049). Age-wise, 60.9% were aged 66 or older, with a similar prevalence of low intake (9.4%) compared to those aged 50–65 (9.6%, *p* = 0.532). Obesity was associated with a lower prevalence (8.7%) compared to non-obese individuals (9.7%, *p* = 0.005). Physical activity showed stronger associations: those not engaging in vigorous activity had a 10.2% prevalence versus 7.9% among active individuals (*p* < 0.001), while moderate activity showed an even starker contrast (12.9% vs. 7.7%, *p* < 0.001). Non-drinkers had a higher prevalence (11.4%) than drinkers (7.5%, *p* < 0.001). High depression levels, low HGS, and stroke were also significantly associated with higher prevalence of low protein intake, with *p*-values all below 0.05. No significant differences were found for heart attack, hip fracture, diabetes, or cancer.

In Table 2 we show the frequency of protein intake from dairy, eggs, and meats among 38,073 respondents. Daily consumption was most common for dairy, with 59.7% reporting intake every day, compared to 30.5% for meats and just 9.9% for legumes/eggs. Weekly intake patterns varied: 47.8% consumed meats 3–6 times per week, while 32.2% did so for legumes/eggs. Less than 10% of respondents consumed any of the three food groups less than once per week. The frequency values were transformed into daily fractions, ranging from 0.0714 for rare intake to 1.0 for daily. Protein weights per 100 g were 8 g for dairy, 12 g for eggs, and 20 g for meats.

**Figure 1:**
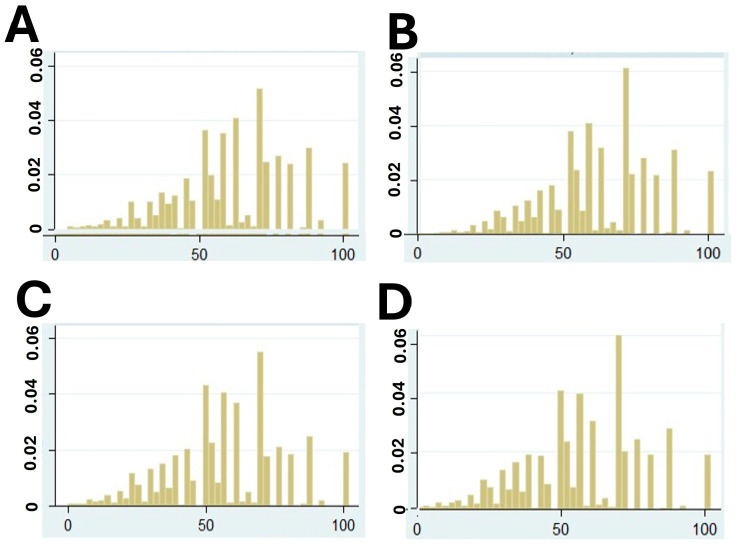
Weighed protein intake frequency (0–100) among men (**A**,**B**) and women (**C**,**D**), aged 50─65 (**A**,**C**) or more than 66 (**B**,**D**) years in the Wave 8 of Survey of Health, Ageing and Retirement in Europe (SHARE).

Figure 1 displays the distribution of weighted protein intake frequency (scaled from 0 to 100) across four demographic groups based on gender and age, using data from Wave 8 of the SHARE survey. Panels A and B represent men aged 50–65 and 66+, respectively, while Panels C and D show the same age groups for women. Among men aged 50–65 (Panel A), the distribution is relatively symmetric, with most individuals clustered around the mid-range of the protein intake index. In contrast, older men (Panel B) show a slight leftward shift, indicating a modest decline in protein intake with age. For women, the distributions are more skewed. Women aged 50–65 (Panel C) already show a tendency toward lower intake frequencies compared to men, with a noticeable concentration in the lower half of the index. This trend becomes more pronounced in women aged 66 and older (Panel D), where the distribution is clearly left-skewed, suggesting a larger proportion of women in this group have low protein intake.

Table 3 presents the distribution of protein intake frequencies across three food groups—dairy, legumes/eggs, and meats—by educational attainment, distinguishing between individuals with low and high education levels. The sample includes 35,653 individuals with low education and 2420 with higher education. For dairy, daily consumption was slightly more common among the low-educated group (60.0%) compared to the highly educated (56.5%), while 3–6 times per week was more frequent among the highly educated (25.7% vs. 22.4%). The *p*-value of 0.003 indicates a statistically significant difference in dairy consumption patterns by education. For legumes and eggs, the differences were more pronounced. Daily consumption was reported by 11.9% of the highly educated versus 9.8% of the low-educated. Moreover, 37.3% of the highly educated consumed these foods 3–6 times per week, compared to 31.9% of the low-educated. The *p*-value here was <0.001, suggesting a significant association between education and intake frequency. Meat consumption also varied: 33.1% of the highly educated consumed meat daily, compared to 30.3% of the low-educated. Interestingly, the highly educated were slightly more likely to consume meat 3–6 times per week (48.2% vs. 47.8%). The *p*-value of <0.001 confirms significant differences in meat consumption by education level.

In Table 4 we present odds ratios (ORs) for ten physical limitations associated with low protein intake—defined as being in the lowest decile of protein frequency—stratified by gender and age group (50–65 years and 66+) and adjusted for country of residence. The results highlight how low protein intake correlates functional impairments, particularly among older adults and women. For low HGS, low protein intake was significantly associated with higher odds in all groups except younger women. The OR was 1.393 for men aged 50–65 and 1.351 (*p* < 0.001) for men 66+, while for women 66+, the OR was 1.213 (*p* < 0.01). Walking 100 m showed significant associations across all groups, with ORs ranging from 1.250 * (*p* < 0.05) in older men to 1.534 * in younger men and 1.508 ** (*p* < 0.01) in younger women, indicating a consistent link between low protein intake and mobility limitations. For stooping, kneeling, or crouching, the effect was significant only among women, with ORs of 1.200 * (50–65) and 1.189 ** (66+), suggesting gender-specific vulnerability in musculoskeletal function. Reaching above shoulder level was also more difficult for those with low protein intake, especially women aged 50–65 (OR = 1.329 *) and men in both age groups (ORs ~1.23). Tasks requiring strength, such as pulling or pushing large objects, were significantly affected in men, with ORs of 1.443 * (50–65) and 1.206 * (66+), while the association was not significant in women. For bathing or showering, older men with low protein intake had significantly higher odds of difficulty (OR = 1.472 **), while other groups showed elevated but insignificant ORs. A striking finding was observed in toileting difficulties, where women aged 50–65 with low protein intake had an OR of 2.272 **, indicating more than double the odds of difficulty compared to their higher-protein peers. Finally, shopping for groceries was significantly impacted in women, with ORs of 1.651 ** (50–65) and 1.221 * (66+), while men showed no significant associations.

## 4. Discussion

This extensive, multi-country analysis of older European adults from the SHARE dataset found that lower habitual protein intake was independently associated with greater odds of reduced HGS and difficulties in performing mobility-related activities. Associations between protein intake and HGS were stronger among men, while functional limitations, such as walking 100 m, stooping, kneeling, extending the arm above the shoulders, and shopping for groceries, appeared more prevalent among women. These findings indicate an association between habitual protein intake and short-term changes in muscle strength and functional outcomes, rather than evidence of long-term progression or causal effects.

The association between low protein intake and reduced HGS supports a substantial body of evidence linking dietary protein adequacy to muscle function in aging populations [17,18]. HGS reflects the overall integrity of the musculoskeletal and neuromuscular systems, serving as a reliable marker of frailty and mortality risk [3]. The individuals with higher protein intake maintain better muscle function and experience slower declines in strength over time [12]. Our findings show a persistence of this relationship across diverse European settings, which aligns with the data from the Newcastle 85+ Study [16].

Most previous cohort studies examining protein intake and functional outcomes estimate absolute intake in grams per kilogram per day using food frequency questionnaires [12,16]. In contrast, the present study applies a frequency-based composite index to rank habitual consumption of major protein-rich food groups, which should be interpreted as a pragmatic proxy rather than a measure of absolute protein intake.

Mechanistically, inadequate protein intake disrupts the balance between muscle protein synthesis and degradation, promoting sarcopenia and weakness [7]. The aging process is accompanied by “anabolic resistance,” in which older muscle responds less efficiently to amino acid stimulation. Diets low in essential amino acids, especially leucine, blunt mTORC1 signalling and limit myofibrillar repair [7]. Consequently, individuals with an inadequate daily protein consumption are more likely to experience muscle atrophy and loss of functional reserve [19]. The consistency of these mechanisms with our findings underscores the biological plausibility of the observed associations.

The relationship between low protein intake and reduced HGS was stronger in men, whereas women exhibited a higher likelihood of mobility difficulties. These differences may reflect sex-specific variations in muscle mass, hormonal profiles, and activity patterns [20]. Men with a higher baseline lean mass may experience greater measurable strength loss when protein intake is insufficient. In contrast, women with lower muscle mass may exhibit functional limitations earlier. Furthermore, older European women often consume smaller meals and fewer protein-rich foods than men, which contributes to their lower total intake [21]. Such gender-related disparities should be considered in nutritional guidelines and public health interventions aimed at promoting healthy aging.

Participants aged 66 years and above demonstrated stronger associations between low protein intake and mobility limitations compared with those aged 50–65 years. This pattern is consistent with reports that the benefits of protein become more evident with advancing age, due to declining appetite, hormonal changes, and chronic inflammation [22]. Older adults often experience diminished absorption of amino acids, intensifying the risk of protein insufficiency even when intake appears adequate [19]. These factors collectively explain why the oldest participants in our study were most vulnerable to the adverse effects of low protein consumption.

Our findings also link low protein intake to broader impairments in functional performance. Walking speed, stair climbing, and balance require coordination, neural control, and energy metabolism. Protein deprivation can compromise all of these elements by limiting substrate availability for mitochondrial function and neurotransmitter synthesis [7]. The consistent associations across diverse measures of physical performance, therefore, likely reflect a network of interrelated muscular and neural pathways rather than an isolated effect on muscle mass.

The sociodemographic correlates of protein intake observed in this study align with findings from other European surveys. Participants with higher educational attainment were more likely to report frequent consumption of protein-rich foods such as dairy, legumes, and lean meats [23]. The descriptive finding that obesity was associated with a lower prevalence of low protein intake should be interpreted with caution. Higher body weight does not necessarily indicate better muscle health and may coexist with reduced muscle mass or strength, a condition commonly described as sarcopenic obesity, which is associated with poor physical function and adverse outcomes in older adults [24]. Education enhances nutritional literacy and influences dietary choices while also correlating with higher socioeconomic status and food security [23]. Public-health efforts aimed at improving nutritional knowledge and access to affordable protein sources could therefore help narrow these disparities.

The quality and source of protein also deserve attention. For example, plant proteins generally have lower digestibility and leucine content compared to animal sources [8]. However, dietary patterns that combine legumes, cereals, and dairy can provide a complete amino acid profile. Encouraging mixed diets tailored to cultural preferences, such as Mediterranean-style or Nordic diets, may offer practical frameworks for achieving sufficient protein quality without excessive animal consumption. Additionally, physical activity interacts synergistically with protein intake in determining muscle outcomes. Experimental evidence consistently shows that resistance training enhances the efficacy of dietary protein on muscle-protein synthesis and physical function [9]. Therefore, public health interventions combining nutritional optimization with accessible community-based strength programs could deliver the most significant benefits for maintaining independence in older adults.

This study has several strengths. It draws upon the extensive SHARE dataset, which encompasses multiple European countries with harmonized representative data collection and large sample sizes, thereby reducing uncertainly about protein intake effects [14]. The use of standardized measures of HGS and functional ability provides a reliable basis for outcome assessment, while stratified analyses by age and gender yield insights into subgroup-specific vulnerabilities. The findings are further strengthened by adjustment for a range of sociodemographic and health-related confounders.

Nonetheless, certain limitations must be acknowledged. Dietary intake was self-reported using food-frequency data rather than quantitative nutrient analysis, which may introduce misclassification bias. The protein intake index was not validated against quantitative dietary methods that estimate intake in grams per kilogram per day. Future validation against detailed dietary assessments or nutritional biomarkers in independent cohorts would strengthen its interpretability and generalizability. The follow-up period between Wave 8 and Wave 9 was relatively short, limiting the ability to capture long-term processes such as sarcopenia and progressive functional decline. The results therefore reflect short-term associations with incident self-reported functional difficulties rather than longitudinal trajectories. Physical activity and protein quality were not jointly modeled with protein intake in the main analyses, despite their well-established interaction in muscle health. This limits the ability to disentangle independent and synergistic effects and should be considered when interpreting the findings. The observational nature of the study precludes causal inference, and unmeasured factors, such as inflammation or subclinical illness, may confound the observed associations. Attrition bias is also possible, as frailer individuals are less likely to participate in subsequent SHARE waves. Moreover, HGS may not fully capture lower-limb performance or muscle power, which are equally crucial for mobility. Most functional outcomes in this study were based on self-reported difficulties rather than objectively measured performance tests, which may introduce bias and misclassification, although such measures are widely used and validated for large-scale epidemiological studies of aging. Reverse causality cannot be excluded, as low protein intake may represent a marker of underlying vulnerability rather than a causal determinant of functional decline. The regression models were adjusted for age, gender, and country of residence; however, residual confounding by factors such as detailed dietary characteristics, socioeconomic circumstances, or health-related behaviors cannot be excluded due to data availability and harmonization constraints within SHARE. Dietary assessment was limited to self-reported consumption frequency of selected protein-rich food groups and did not include detailed nutritional evaluation and should be interpreted as reflecting habitual dietary patterns rather than isolated effects of protein intake. Furthermore, given the observational design, protein intake may act as a marker of broader health and functional vulnerability, with associations potentially influenced by underlying factors including appetite, comorbidity burden, physical activity, and socioeconomic conditions. Future analyses incorporating objective dietary biomarkers and longitudinal follow-up could help address these limitations.

## 5. Conclusions

Altogether, this study demonstrates that lower habitual protein intake is associated with a higher likelihood of short-term incident muscle weakness and functional difficulties among older European adults. These findings suggest that habitual protein intake may play a contributory role in maintaining physical function in aging populations. Future SHARE waves and experimental studies should aim to validate these findings using biomarker-based dietary measures and to explore whether targeted protein supplementation, when combined with structured physical activity, can prevent or reverse functional decline. Policies that integrate nutrition education, economic accessibility, and physical activity promotion could substantially improve the quality of life for Europe’s aging population.

## Figures and Tables

**Table 1:** Sample characteristics by protein intake based on the threshold for the recommended daily protein intake for European older adults. The data for gender, age, and protein intake are taken from the base year 2018 (SHARE wave 8). All the other variables representing mental health or disease are from the subsequent year 2021-22 (SHARE wave 9). The first characteristic for any variable is the reference group. (SHARE; Survey of Health, Ageing and Retirement in Europe, HGS; Handgrip Strength).

		Share of Respondents	Prevalence of Low Protein	Normal Protein	Low Protein	Sample	
		%	Intake, %	Intake, n_1_	Intake, n_2_	Size, n	*p*-Value
Gender	Male	42.5	9.8	14,602	1593	16,195	0.049
	Female	57.5	9.2	19,857	2021	21,878	
Age	50–65	39.1	9.6	13,451	1430	14,881	0.532
	66+	60.9	9.4	21,008	2184	23,192	
Obese	No	75.7	9.7	26,028	2806	28,834	0.005
	Yes	24.3	8.7	8431	808	9239	
Physical activity: Vigorous	No	69.6	10.2	23,796	2697	26,493	0.000
	Yes	30.4	7.9	10,663	917	11,580	
Physical activity: Moderate	No	34.6	12.9	11,470	1694	13,164	0.000
	Yes	65.4	7.7	22,989	1920	24,909	
Drinks alcohol	No	51.8	11.4	17,490	2244	19,734	0.000
	Yes	48.2	7.5	16,969	1370	18,339	
High depression level	No	83.1	8.7	28,875	2748	31,623	0.000
	Yes	16.9	13.4	5584	866	6450	
Low HGS	No	84.3	8.9	29,240	2872	32,112	0.000
	Yes	15.7	12.4	5219	742	5961	
Heart attack	No	86.5	9.4	29,813	3110	32,923	0.439
	Yes	13.5	9.8	4646	504	5150	
Stroke	No	95.5	9.4	32,930	3427	36,357	0.042
	Yes	4.5	10.9	1529	187	1716	
Hip fracture	No	98.2	9.5	33,847	3541	37,388	0.294
	Yes	1.8	10.7	612	73	685	
Diabetes	No	84.5	9.5	29,111	3048	32,159	0.823
	Yes	15.5	9.6	5348	566	5914	
Cancer	No	94.1	9.5	32,417	3420	35,837	0.175
	Yes	5.9	8.7	2042	194	2236	
Total		100	9.5	34,459	3614	38,073	

**Table 2:** Frequency of protein intake from different food items from Wave 8 of the Survey of Health, Ageing and Retirement in Europe (SHARE).

	Dairy	Legumes, Eggs	Meats	Calculation Weight, Frequency
Less than once/week	4.1	7.8	2.1	0.0714
Once/week	4.0	19.4	4.8	0.1429
Twice/week	9.6	30.6	14.8	0.2857
3–6 times/week	22.6	32.2	47.8	0.6429
Every day	59.7	9.9	30.5	1.0000
Total	100	100	100	
Calculation weight, food type	8	12	20	

**Table 3:** Distribution across protein intake frequencies by food type and educational level from Wave 8 of the Survey of Health, Ageing and Retirement in Europe (SHARE). Higher education is defined as 14–18 years of education.

		Education		Education		
		Low	High	Low	High	
		n	n	%	%	*p*-Value
Dairy	Less than once/week	1454	105	4.1	4.3	0.003
	Once/week	1414	98	4.0	4.0	
	Twice/week	3432	229	9.6	9.5	
	3–6 times/week	7974	621	22.4	25.7	
	Every day	21,379	1367	60.0	56.5	
Legumes, eggs	Less than once/week	2805	180	7.9	7.4	0.000
	Once/week	7001	378	19.6	15.6	
	Twice/week	10,976	671	30.8	27.7	
	3–6 times/week	11,374	903	31.9	37.3	
	Every day	3497	288	9.8	11.9	
Meats	Less than once/week	734	58	2.1	2.4	0.000
	Once/week	1731	103	4.9	4.3	
	Twice/week	5348	292	15.0	12.1	
	3–6 times/week	17,025	1167	47.8	48.2	
	Every day	10,815	800	30.3	33.1	
Total		35,653	2420	100	100	

**Table 4:** Odds ratios for developing various physical difficulties (SHARE Wave 9) associated with the lowest docile of protein intake frequency (SHARE Wave 8), adjusted for country and stratified analyses by age group and gender. A low protein intake was defined as being in the lowest 10% (bottom decile) of the protein frequency index within the given age and gender groups. All estimates reflect comparisons between participants in the lowest 10% of the protein intake index and those with higher protein intake within each age and sex stratum, * *p* < 0.05, ** *p* < 0.01, *** *p* < 0.001. (SHARE; Survey of Health, Ageing and Retirement in Europe, HGS; Handgrip Strength).

	Males:		Females:		All:
Outcome:	50–65 Years	> = 66 Years	50–65 Years	> = 66 Years	Sample Size, n
HGS (low)	1.393 *	1.351 ***	1.129	1.213 **	38,073
Rising from the chair	1.136	1.165	1.163	1.160 *	38,073
Walking 100 m	1.534 *	1.250 *	1.508 **	1.341 ***	38,073
Climbing stairs	1.117	1.132	1.181	1.057	38,073
Stooping, kneeling, crouching	0.971	1.081	1.200 *	1.189 **	38,073
Reaching or extending arms above the shoulder	1.229	1.232	1.329 *	1.192 *	38,073
Pulling or pushing large objects	1.443 *	1.206 *	1.227	1.112	38,073
Bathing or showering	1.414	1.472 **	1.506	1.127	38,073
Using the toilet, including getting up or down	1.478	1.333	2.272 **	1.174	38,073
Shopping for groceries	1.250	1.047	1.651 **	1.221 *	38,073
Sample size, n	6104	10,091	8777	13,101	38,073

## Data Availability

The data is publicly available after application from https://share-eric.eu/. Access to data requires an individual to register freely, followed by the acceptance of the SHARE Conditions and signing the SHARE User Statement. After approval of these documents, data can be downloaded using the personal ID and password.
